# Brain Surface Area Alterations Correlate With Gait Impairments in Parkinson’s Disease

**DOI:** 10.3389/fnagi.2022.806026

**Published:** 2022-01-27

**Authors:** Xuan Wei, Zheng Wang, Mingkai Zhang, Min Li, Yu-Chen Chen, Han Lv, Houzhen Tuo, Zhenghan Yang, Zhenchang Wang, Fang Ba

**Affiliations:** ^1^Department of Radiology, Beijing Friendship Hospital, Capital Medical University, Beijing, China; ^2^Department of Neurology, Beijing Friendship Hospital, Capital Medical University, Beijing, China; ^3^Clinical Epidemiology and EBM Unit, National Clinical Research Center for Digestive Diseases, Beijing Friendship Hospital, Capital Medical University, Beijing, China; ^4^Department of Radiology, Nanjing First Hospital, Nanjing Medical University, Nanjing, China; ^5^Division of Neurology, Department of Medicine, University of Alberta, Edmonton, AB, Canada

**Keywords:** surface area, structural magnetic resonance imaging, Parkinsion’s disease (PD), surface-based morphometry (SBM), gait impairment

## Abstract

Parkinson’s disease (PD) is a common neurodegenerative disease with progressive gait, cognition, and overall functional decline. Surface area changes are frequently seen with aging. In neurodegenerative diseases, the changes can be evident with disease progression. The current study aimed to study the regional microstructural alterations using surface-based morphometry to correlate with gait measures of the pace and rhythm domains in PD patients. We hypothesize that specific regional surface changes can be associated with PD gait impairments. Surface analysis might provide a useful tool for assessing PD for functional status and specific motor domains, such as gait in PD, and potentially could serve as an imaging marker in conjunction with other imaging markers. Twenty-seven PD patients and 37 healthy controls were included. The clinical assessment included Mini-Mental State Exanimation, PD motor assessment, clinical gait testing, and objective/quantitative gait assessment. For patients with PD, all motor and gait testing were performed during both OFF and ON medication states. Three Tesla MRI and high-resolution 3D structural images were acquired with an MP-RAGE pulse sequence. Structural image data preprocessing was performed using the DPABISurf toolbox. Clinical characteristics between PD and control group were compared, and correlation between the surface area and behavioral data were analyzed. At the left lateral temporal cortex (LTC) and right inferior parietal cortex (IPC), PD patients have significantly larger surface areas when compared to controls (*P* < 0.05) using surface-based morphometry. The surface area changes of the left LTC and right IPC were associated with the worse performance of gait assessed by Berg Balance Scale and Timed Up and Go during OFF (*P* < 0.01). The left LTC area changes significantly correlated with the number of steps, velocity, and the stride length of the pace domain in the ON state. Our findings suggest that PD is associated with a characteristic regional pattern of larger surface area in the left LTC and right IPC. These regional changes were associated with the pace domain of the gait in the ON state. Overall, surface-based analyses might provide a useful tool for assessing PD for functional status and specific motor domains, such as gait in PD, and potentially could serve as an imaging marker.

## Introduction

Parkinson’s disease (PD) is a common neurodegenerative disease. The prevalence increases with age. PD pathology affects multiple neural circuitries. Gait and balance impairments occur more frequently as the disease progresses, and are usually more disabling than many other motor symptoms. The detailed pathophysiology of gait impairment in PD, including the freezing of gait (FOG) is not fully understood. In brief, gait control requires the activation of the entire nervous system and musculoskeletal system, including the spinal locomotor network, mesencephalic locomotor region (MLR), basal ganglia output to MLR, and motor control in the frontal lobe (Takakusaki, 2013). In addition, the parietofrontal connection and the corticoreticulospinal pathway also contribute to gait control given its role in anticipatory postural control. Multisensory input provides cognitive and emotional references to the cerebral cortex and limbic system, to guide voluntary movements or emotional motor behavior depending on the context (Takakusaki, 2013). In PD, although the motor symptoms are thought to be a result of the loss of dopaminergic neurons in the substantia nigra pars compacta (SNc), PD is more complex than a dopamine-responsive motor disorder, particularly in its advanced stages. Widespread neuronal loss is evident in the locus coereleus, the nucleus basalis of Meynert, the dorsal motor nucleus of the vagus nerve, the pedunculopontine nucleus, the raphe nuclei, the hypothalamus, and the olfactory bulb (Giguere et al., 2018). Other non-dopaminergic neurotransmitter systems are affected, such as the cholinergic, noradrenergic, serotonergic, adenosinergic, glutamatergic, GABAergic, etc. (Kalia et al., 2013). Cognitive decline (likely secondary to the cholinergic deficiency and brain atrophy) is a common non-motor feature of PD and can be very disabling (Delgado-Alvarado et al., 2016; Aarsland et al., 2017). The relationship between gait and cognitive decline has been observed in PD patients as their poor cognitive function contributes to worse gait, making them more prone to falls (Camicioli et al., 1998).

Gait can be modeled as a combination of five domains: pace, rhythm, variability, asymmetry, and postural control (Lord et al., 2013). Abnormal gait and postural control can produce FOG, which frequently leads to falls, and is the main reason for loss of life independence and increased mortality. In PD, the risk factors for FOG include longer duration of disease and no systematic treatment, suggesting that FOG can be related to the degree of dopaminergic neuron loss (Camicioli et al., 1998). Neuroimaging is a non-invasive tool to analyze brain structure and function. The recent advances in imaging techniques allow the assessment of gross and microstructural changes of the brain in neurodegenerative diseases. Such assessment in relation to clinical parameters can potentially serve as biomarkers to monitor disease progression. Dysfunction in each of the gait domains may correlate with specific cognitive domains corresponding to specific cortical areas (Shine et al., 2013). Among the imaging modalities, high-resolution three-dimensional (3D) structural T1-weighted imaging technology with whole brain structure information of each brain region is a valid tool for morphological analysis of the brain (Sartoretti et al., 2020).

The two commonly used methods to measure brain morphological changes with structural MRI are voxel-based morphometry (VBM) and surface-based morphometry (Winkler et al., 2010). VBM is a volume-level analysis, which can reconstruct regions of interest or the entire brain through 3D T1 weighted imaging, and can extract cortical and subcortical measurements, thus quantifying the volume of brain tissue at the voxel level (Ashburner and Friston, 2000). Volume-based analysis has been widely used. In PD research, VBM has been applied in cortical thickness, surface area, and subcortical volume assessment (Gerrits et al., 2016; Wilson et al., 2019; Fang et al., 2020). Previous studies have shown changes in cortical and subcortical gray matter in PD-FOG individuals, such as thinning of the cortex, or decreased volume. In more advanced disease, PD patients exhibit progressive cortical thinning and subcortical volume loss which correlated with the development of cognitive impairment (Wilson et al., 2019). Kostic et al. studied the degree and distribution of cortical atrophy and showed that PD-FOG patients had frontal and parietal cortical atrophy, and the severity of FOG correlated with the degree of the bilateral atrophy in these regions (Kostic et al., 2012). VBM has also been applied in PD (Kobayakawa, 2017), in non-dementia PD patients, left anterior cingulate atrophy was observed when compared with healthy controls (HCs; Summerfield et al., 2005).

Cortical involvement is well documented in PD as the disease progresses (Braak et al., 2003). Surface-based morphometry extracts and analyzes various brain features based on the characteristics of the cerebral cortex (Yun et al., 2013), including curvature (Van Essen, 2005), thickness (Wei et al., 2021), sulcal depth (Kippenhan et al., 2005) and area (Storsve et al., 2014). A previous study in normal aging individuals combining volume-based and surface-based methods to measure cortical thickness and surface area found that the surface-based method yielded higher sensitivity, although overall results showed the same trend (Hutton et al., 2009). Cortical thickness has been used as a sensitive measure for gray matter loss in Lewy body spectrum disorders (Watson et al., 2015), and negative correlations exist between the cortical thickness of occipital areas and performance on a verbal memory task (Gerrits et al., 2016). In a large study, Sheng et al. reported that compared with 1,172 healthy controls, no significant cortical thickness alterations were seen in patients with PD, questioning the validity of the cortical thickness as an imaging marker for PD (Sheng et al., 2021). In addition, other findings indicated significantly increased cortical areas in PD patients with depression in the orbitofrontal regions and insula when compared with those without depression (Huang et al., 2016). The results of the studies on cortical thickness are somewhat inconsistent, but so far there is far less research on the surface area of the brain in PD patients (Pietracupa et al., 2018; Zhang et al., 2021), and only limited data on quantitative gait assessment examining velocity, cadence, variability, asymmetry, and postural control, especially in the context of structural changes in PD (Surkont et al., 2021). Therefore, studying the quantitative gait parameter in the context of brain surface area can provide more insight into PD gait impairment.

In the current study, our goal is to evaluate how regional surface area change of the brain correlates with gait performance in PD patients. We performed clinical assessments, including PD motor examination and gait measures during both ON and OFF states in PD participants, and aimed to explore the relationship between dopamine-responsive and non-responsive gait impairment and to correlate with MR changes in PD. Specifically, we aimed to determine the extent to which regional microstructural alterations correlate with gait measures focusing on the pace and rhythm domains of gait. Systematical studies on the relative changes at the surface-based structural level in PD, and correlating such changes with clinical parameters can provide more insight into the mechanism of gait impairment in PD. The current study addresses an emerging topic since such assessments in relation to clinical parameters can potentially serve as biomarkers to monitor disease progression, particularly in gait impairment in PD.

## Materials and Methods

### Standard Protocol Approval, Registration, and Patient Consent

This study was approved by the ethics committees of Beijing Friendship Hospital, Capital Medical University, 2019-P2-283-02. All participants provided written informed consent prior to participation.

### Patients Enrollment

Patients with PD receiving optimal treatment with dopaminergic medications were recruited from the Movement Disorders Program at Beijing Friendship Hospital of Capital Medical University. All PD participants were assessed by movement disorders neurologists who confirmed the diagnosis of idiopathic PD based on UK Parkinson’s Disease Society Brain Bank criteria (Hughes et al., 2002). The exclusion criteria were: atypical or secondary Parkinsonism; confounding medical or psychiatric condition(s); any condition that prevents the ability to give informed consent; and other neurological diseases leading to motor deficit. Community volunteers without neurological and/or psychiatric disorders were recruited as HCs. Twenty-seven patients and 37 HCs were enrolled from April 2020 to March 2021. Demographics and clinical status are listed in Table 1. Patients with gait impairment were identified from the history and on the International Parkinson and Movement Disorder Society Unified Parkinson’s Disease Rating Scale (MDS-UPDRS) part-II, and III (a score ≥1 on 2.12, 2.13 on MDS-UPDRS-II or a score ≥1 on items 3.10 or 3.11 on MDS-UPDRS-III). PD participants were assessed with the following tests to characterize disease status and gait impairment: full MDS-UPDRS, Berg Balance Scale (BBS), Dynamic Gait Index, New Freezing of Gait Questionnaire (nFOGQ), and the Timed-Up and Go (TUG). These tests have been shown to be valid tools in PD assessment.

**Table 1:** Demographic and clinical characteristics of the control and PD participants.

Measurements	Control	PD		
		PD Clinical parameters		
		ON State		OFF State
Age	57.0 (51.0–65.0)		68.0 (63.0–70.0)**	
Sex (% Male)	10 (37.74)		15 (40.54)	
Education (> 9year)	25 (67.57)		20 (74.07)	
Duration of disease (months)	n/a		48.0 (28.0–84.0)	
MMSE	27.0 (25.0–28.0)		28.00 (28.0–29.0)	
MASE	11.0 (7.0–17.0)		12.00 (5.0–17.0)	
BAI	25.0 (22.0–26.0)		26.0 (24.0–29.0)**	
BDI	3.0 (1.0–5.0)		7.0 (4.0–10.0)**	
LEDD (mg)	n/a		550.0 (450.0- 725.0)	
NFOGQ	n/a		11 (40.74)	
MDS-UPDRS-III	n/a	16.0 (13.0–23.0)		29.0 (20.0–36.0)^#^
MDS-UPDRS Total	n/a	39.0 (26.0–47.0)		47.0 (35.0–61.0)^#^
TUG	8.17 (7.35–8.82)	9.95 (8.47–11.89)**		10.92 (9.02–13.93)**
BBS	56.0 (55.0–56.0)	52.0 (46.0–54.0)**		49.0 (39.0–52.0)**
Velocity SSP (cm/s)	123.46 (115.32–130.29)	104.41 (79.30–115.77)**		99.86 (78.86–109.35)**
Cadence SSP (step/min)	113.31 (107.64–121.01)	114.50 (105.51–119.18)		113.88 (103.32–120.53)
Stride time SSP (s)	1.05 (0.99–1.11)	1.04 (1.01–1.12)		1.05 (1.00–1.16)
Stride length SSP (cm)	129.38 (119.66–139.21)	107.13 (95.60–124.36)**		105.50 (83.21–115.82)**
Velocity FP (cm/s)	151.26 (144.33–168.62)	125.59 (104.28–141.53)**		121.97 (98.18–137.26)**^#^
Cadence FP (step/min)	127.21 (118.03–135.34)	120.73 (115.12–130.74)		124.66 (112.06–132.04)
Stride time FP (s)	0.95 (0.89–0.98)	0.99 (0.91–1.04)		0.96 (0.91–1.07)
Stride length FP (cm)	143.21 (130.64–156.61)	122.25 (105.02–138.49)**		118.68 (93.31–127.12)**^#^

PD, Parkinson’s disease; MMSE, Mini-Mental State Examination; MAES, Modified Apathy Evaluation Scale; LEDD, levodopa equivalent daily dose (mg); nFOGQ, New Freezing of Gait Questionnaire; MDS-UPDRS, International Parkinson and Movement Disorder Society-Unified Parkinson’s Disease Rating Scale; MDS-UPDRS-III, MDS-UPDRS motor score; TUG, Timed up and Go Test; BBS, Berg Balance Scale; SSP, self-selected pace; FP, fast pace.For control, there is no ON or OFF state. Same set of clinical data from the control was used to analyze against PD group. **P < 0.01, compared with control group. ^#^*P* < 0.05, compared with ON State.

The clinical assessments were performed in the “defined OFF” state, which is 12 h after their last PD medications, and ON state in the same day with a supra-ON dose of levodopa (125% of the morning dose of LED; Tomlinson et al., 2010) after participants reported ON when medications kicked in, since many PD participants experienced motor fluctuations. If dose failure occurred, patients were allowed to take another dose after 1 h. Patients were examined by a movement disorders neurologist during the defined OFF and ON time, and MDS-UPDRS score was documented. For all PD and control participants, objective gait assessments were assessed using a 20-foot-long computerized Zeno Walkway system (Proto Kinetics, Havertown, PA, USA) at a self-selected pace (SSP) and fast pace (FP). Mini-Mental State Examination (MMSE; Folstein et al., 1975) was used to test cognition for all participants. Improvement in gait measure from “OFF” state to “ON” state in the relevant domains on MDS-UPDRS and with objective gait testing indicated a dopamine-responsive gait improvement. As co-morbidity anxiety and depression are widely reported, the Beck Depression Inventory (BDI) score, Beck Anxiety Inventory (BAI) score and Modified Apathy Evaluation Scale (MAES) were used to assess the presence of co-morbid mood and anxiety disorders (Table 1).

### MRI Imaging Protocol

#### Data Acquisition

Images were obtained using a 3.0 T MRI system (Prisma, Siemens, Erlangen, Germany) with a 64-channel phase-array head coil. Imaging studies were performed at the Medical Imaging Research Center of Beijing friendship hospital. High-resolution 3D structural T1-weighted images were acquired using a 3D magnetization-prepared rapid gradient echo (MP-RAGE) sequence with the following parameters: *FA* = 7°, inversion time (TI) = 1,100 ms, slice thickness = 1 mm, repetition time (TR) = 2530 ms, echo time (TE) = 2.98 ms, number of slices = 192, field of view (FOV) = 256 mm × 224 mm, and matrix = 256 × 224, bandwidth = 240 Hz/Px, resulting in an isotropic voxel size of 1 mm × 1 mm × 1 mm.

#### Anatomical Data Preprocessing

Data preprocessing was performed using DPABISurf^1^, which is a surface-based resting-state fMRI data analysis toolbox (Chen and Yan, 2021). HCP template is a built-in template in it, used to divide the cortex. The results included in this manuscript come from preprocessing performed by DPABISurf using fMRIPrep 20.0.5 (Esteban et al., 2019), which is based on Nipype 1.4.2 (Gorgolewski et al., 2011). The T1-weighted (T1w) image was corrected for intensity nonuniformity (INU) with N4BiasFieldCorrection (Tustison et al., 2010), distributed with ANTs 2.2.0 (Avants et al., 2008), and used as the T1w-reference throughout the workflow. The T1w-reference was then skull-stripped with a *Nipype* implementation of the antsBrainExtraction.sh workflow (from ANTs), using OASIS30ANTs as the target template. Brain tissue segmentation of cerebrospinal fluid, white matter (WM) and gray matter was performed on the brain-extracted T1w images using FMRIB’s Automated Segmentation Tool (FSL 5.0.9; Zhang et al., 2001). Brain surfaces were reconstructed using recon-all (Dale et al., 1999), and the previously estimated brain mask was refined with a custom variation of the method to reconcile ANTs-derived and FreeSurfer-derived segmentation of the cortical gray matter of Mindboggle (Klein et al., 2017). Volume-based spatial normalization to one standard space (MNI152NLin2009cAsym) was performed through nonlinear registration with ANTs Registration (ANTs 2.2.0) using brain-extracted versions of both the T1w reference and the T1w template. The following template was selected for spatial normalizatio*n*: ICBM 152 Nonlinear Asymmetrical template version 2009c (Fonov et al., 2009). The surface area was calculated in the above process.

### Statistical Analysis

Demographic and clinical characteristics were described as the percentage for categorical variables and median with interquartile range for continuous variables. Comparisons between PD patients and HCs were made by using the chi-square test for categorical variables and the test for continuous variables. Spearman’s rank correlation was calculated to assess the relationship between the surface area and patient/disease related rating scale (MDS-UPDRS-III scores, MMSE scores, the TUG test scores, and BBS scores). Normality testing revealed that not all clinical and objective gait measure data met normality assumptions.

Objective gait parameters of PD patients and HCs were compared using Wilcoxon rank-sum test. For PD participants, changes in objective gait parameters between ON and OFF state were analyzed by using paired two-sample Wilcoxon rank-sum test. The correlation between surface area and gait parameters was analyzed using a generalized linear model, adjusted for age and sex, respectively in the PD and HCs groups. Standardized regression coefficients (*β*) and their corresponding *P-*value were calculated. The analyses were done using SAS version 9.3 (SAS Inc., Cary, NC, USA).

For the surface area data, a *P-*value from the area analysis less than 0.05 (*P* < 0.025 for each hemisphere) was considered statistically significant (Monte Carlo simulation corrected). *P* < 0.05 was set as the threshold to determine significance. The surface area results were visualized with DPABISurf.

## Results

### Demographics and Behaviors Measures of the Study Participants

Demographic and clinical data are shown in Table 1. The control group is younger than the PD group (*P* < 0.01). There were no significant differences in MMSE or MAES scores between the two groups. The scores of BAI and BDI in the PD group were significantly higher than those in the control group (Table 1). Normality testing revealed that not all clinical and objective gait measure data met normality assumptions (Tables s 1, 3). Compared with the HCs, the score of BBS in the PD group was significantly lower, while the TUG value was higher (Table 1). The velocity and stride length of the HCs were higher than those of PD patients. In the PD group, the ON state improved the velocity and stride length at SSP and FP (Table 1).

**Table 2:** Difference in cortical area of the left and right hemisphere between PD patients and the controls.

Brain regions	HCP	Cluster size (mm)	Coordinates MNI			Peak *F* score
			x	y	z
Left lateral temporal cortex	136	66	−49	−42	−21	4.59
Right inferior parietal cortex	155	140	31	−37	−11	4.59

*Statistically significant differences in the cortical area were defined as *P* < 0.05 (*P* < 0.025 for each hemisphere), Monte Carlo Simulation corrected after correcting for age, sex, education, and the head motion. Abbreviations: MNI, Montreal Neurological Institute; HCP, Human Connectome Project*.

**Table 3:** Analysis of the surface area changes in left LTC and right IPC correlate with the gait changes in Parkinson’s disease using the regression model.

Gait measures	Left LTC	Right IPC
	β/*P* value	β/*P* value
ON
Velocity SSP (cm/s)	0.50/**0.04**	−0.06/0.82
Cadence SSP (step/min)	0.11/0.58	−0.17/0.36
Stride time SSP (s)	−0.13/0.53	0.162/0.40
Stride length SSP (cm)	0.52/**0.03**	0.02/0.92
OFF
Velocity SSP (cm/s)	0.03/0.90	<0.01/1.00
Cadence SSP (step/min)	−0.23/0.27	−0.23/0.23
Stride time SSP (s)	0.29/0.15	0.18/0.34
Stride length SSP (cm)	0.04/0.87	0.06/0.80
ON
Velocity FP (cm/s)	0.63/**0.01**	−0.06/0.80
Cadence FP (step/min)	0.15/0.49	−0.07/0.73
Stride time FP (s)	−0.15/0.48	0.10/0.61
Stride length FP (cm)	0.75/<0.00*	−0.05/0.85
OFF
Velocity FP (cm/s)	<0.01/0.90	0.01/0.96
Cadence FP (step/min)	−0.23/0.20	−0.16/0.40
Stride time FP (s)	0.30/0.14	0.17/0.38
Stride length FP (cm)	0.01/0.96	0.02/0.95

*P = 0.0021; β, Standardized regression coefficients; ON, in medication ON state; OFF, in medication OFF state; SSP, self-selected pace; and FP, fast pace. LTC, lateral temporal cortex; IPC, Inferior parietal cortex. The bold values highlight the significant correlation coefficients between objective gait parameters and the surface area of left LTC.

### Analysis of the Surface Areas

**Figure 1:**
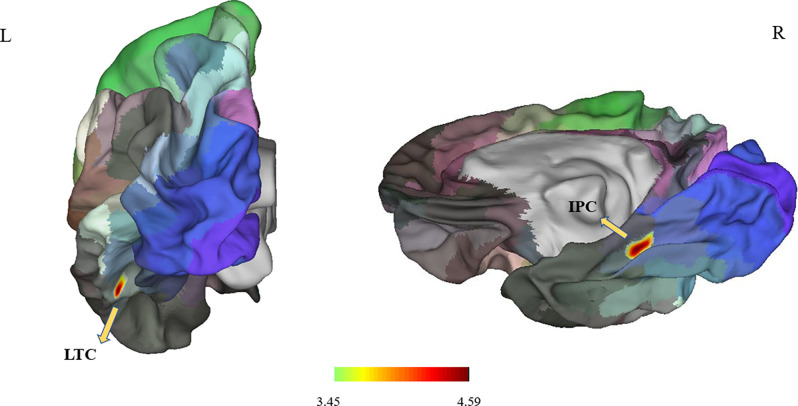
It showed differences in surface area in the left LTC and right IPC (*P* < 0.05; *P* < 0.025 for each hemisphere) corrected by Monte Carlo simulation; L, left; R, right; Abbreviations: LTC, lateral temporal cortex; IPC, right inferior parietal cortex.

Between the PD and the control groups, there were significant differences in surface area of the left lateral temporal cortex (LTC) and the right inferior parietal cortex (IPC; *P* < 0.05 corrected by Monte Carlo simulation; Figure 1, Table 2).

**Figure 2:**
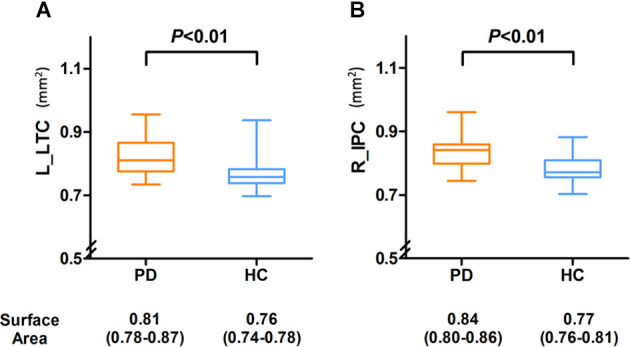
Compared with the PD group, the surface area was significantly decreased in the left LTC **(A)** and right IPC **(B)** of HC group and compared with the HC group, and the PD group demonstrated a slightly larger area in the left LTC and right IPC, and there were also significant differences between the two groups. Abbreviations: L, left; R, right; LTC, lateral temporal cortex; IPC, inferior parietal cortex.

As shown in Figure 2, the surface area of the left LTC and right IPC was larger in the PD patients compared with HCs, and the differences remained after adjusting for age and gender (*P* = 0.002 and *P* = 0.008, respectively).

### Correlation Analysis

**Figure 3:**
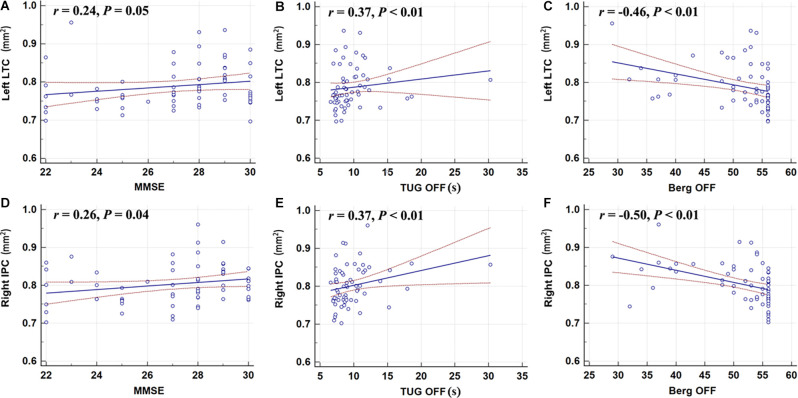
Correlation analyses showed that the left LTC **(A–C)** and the right IPC **(D–F)** were positively correlated with the TUG-off test score; the surface area of the right IPC was weakly correlated with the MMSE score; the left LTC and right IPC were negatively correlated with and BBS-off score (all *P* < 0.01). MMSE, Mini-Mental State Examination; TUG, Timed-Up and Go; BBS, Berg Balance Scale.

The surface area of the right IPC was weakly correlated with the MMSE score (*r* = 0.26, *P* = 0.04); however, the left LTC showed no correlation with the MMSE (Figure 3). The surface area of the left LTC and right IPC were positively correlated with TUG-off scores, and negatively correlated with and BBS-off score (all *P* < 0.01; Figure 3), suggesting a larger surface area of these two regions correlated with worse gait performance during OFF state. There were no correlations between the surface area of the left LTC and right IPC with BDI, BAI, MDS-UPDRS-III, or N-FOGQ.

Using Standardized regression coefficients, the surface-based measures from the left LTC showed the strongest correlation with the objective gait parameters (Table 3). While in the ON state, larger left LTC surface area values were positively correlated with velocity SSP (Table 3, *P* = 0.04) and stride length SSP (Table 3, *P* = 0.03). Larger left LTC surface area values were positively correlated with velocity FP (Table 3, *P =* 0.01) and stride length FP (Table 3, *P* = 0.002). The surface area in the right IPC did not show any significant correlation with quantitative gait parameters (Table 3).

## Discussion

Using surface-based morphometry, we analyzed the changes in surface area in patients with PD. Surface areas were expanded in the left LTC and right IPC. Enlargement of certain cortical surface areas in PD was reported before (Kostic et al., 2012). It has been shown that the enlargement of the surface area is related to the degree of local folding (Jubault et al., 2011). Therefore, one can postulate that cortical folding might universally contribute to surface area (Mota and Herculano-Houzel, 2015). The local surface area may reflect the state of the related white matter fibers (Van Essen, 1997); the greater the tension or contraction of these fibers, the wider the expansion of the cortical surface area. Our current study suggested cortical morphological change may happen in the relatively early course of PD given our patients are mostly in the early to mid-stage of the disease (average Hoehn and Yahr stage 1.75). Given the pathological process involved in increased surface area, both gray matter and white matter changes may co-occur in specific brain regions, and contribute to gait impairment.

Our key finding of the study is that surface area expansion in the left LTC and right IPC were correlated with overall worse gait performance during OFF state as reflected by TUG and BBS. Such correlations help explain the neuropathological basis of gait impairment in PD and are consistent with the dopaminergic neuronal loss (Lee et al., 2011). Considering PD pathology, OFF state measures likely relate to the disease state itself, and are unlikely to change in response to dopaminergic treatment, which is what we observed with TUG and BBS, both did not improve from OFF state to ON state. The left LTC area changes with gait impairments were found in our study. This is consistent with previous evidence showing gait impairment was related to temporal lobe atrophy (Annweiler et al., 2014). Most importantly, we found that the structural changes in the left LTC correlated with gait measures of the pace domain in the ON state only (gait velocity and stride length), suggesting such correlation is dopamine-independent (Surkont et al., 2021).

Although we have observed the surface areas expansion in the left LTC and right IPC, and both regions are critical in cognition and higher-level information processing, we did not reveal a strong correlation with MMSE in the PD group. The fact that most PD patients’ MMSE scores were within the normal range might have explained the lack of correlation between the cognitive test and imaging measures. In addition, we did not perform more detailed neuropsychological testing since it is not the main objective of the study.

The scores of BAI and BDI in the PD group were significantly higher than those in the HCs group. Anxiety and depressive disorders are common in PD, which are associated with structural and functional changes of multiple brain regions (Schrag and Taddei, 2017; Carey et al., 2021). In PD with mild to moderate depression, cortical thickness of the precuneus cortex was also significantly increased (Zanigni et al., 2017). However, in our cohort, we did not observe any significant correlations.

The strengths of the study include detailed clinical assessment, and detailed gait assessment using computerized objective gait measures during both ON and OFF states. We also focused on specific gait domains. There are some limitations to this study. It is a single-centered study with a relatively small sample size. Secondly, we did not apply volume-based analysis to further explore the structural characteristics of PD in conjunction with the surface analysis for a more complete picture. However, our findings are still valid and can provide new insight into the field. In future studies, we will combine other structural and functional surface-based indicators, such as ReHo, degree centrality, fALFF, to gain a more comprehensive and complete understanding of the PD pathology, and aim to establish a more reliable imaging portfolio for PD gait impairment.

## Conclusion

Our findings suggest that PD is associated with a regional surface area change—a larger surface area alteration in the LTC and IPC. These changes in surface area may suggest a combined gray matter and white matter dysfunction. Moreover, the regional microstructural integrity changes in the left LTC were associated with the pace domain of the gait. Overall, surface area analyses might provide a useful tool for assessing PD motor function, such as gait impairments. In conjunction with other biochemical markers and clinical biomarkers, such imaging tools potentially could serve as an imaging marker, and contribute to a more complete and reliable multi-module biomarker profile for PD diagnosis and prognosis.

## Data Availability Statement

The original contributions presented in the study are included in the article, further inquiries can be directed to the corresponding author/s.

## Ethics Statement

The studies involving human participants were reviewed and approved by the Ethics Committees of our Research Institution (Beijing Friendship Hospital, Capital Medical University, 2019-P2-283-02). The patients/participants provided their written informed consent to participate in this study. Written informed consent was obtained from the individual(s) for the publication of any potentially identifiable images or data included in this article.

## Author Contributions

XW: research project—execution; statistical analysis—design and execution; and manuscript—writing of the first draft. ZW and MZ: research project—execution; statistical analysis—execution; and manuscript—review and critique. ML and Y-CC: statistical analysis—design, execution, review and critique. HL and HT: research project—organization and execution; manuscript—review and critique. ZY: research project—organization and execution. ZW: research project—conception and organization. FB: research project—conception and organization; statistical analysis—design, review and critique; manuscript—review and critique. All authors contributed to the article and approved the submitted version.

## Conflict of Interest

The authors declare that the research was conducted in the absence of any commercial or financial relationships that could be construed as a potential conflict of interest.

## Publisher’s Note

All claims expressed in this article are solely those of the authors and do not necessarily represent those of their affiliated organizations, or those of the publisher, the editors and the reviewers. Any product that may be evaluated in this article, or claim that may be made by its manufacturer, is not guaranteed or endorsed by the publisher.

## Footnotes

^1^ http://rfmri.org/DPABISurf
